# Analysis of the geographic pattern of the police reports for domestic violence in Girona (Spain)

**DOI:** 10.1186/s12889-022-12916-4

**Published:** 2022-03-21

**Authors:** Laura Serra, Laura Vall-llosera, Diego Varga, Carme Saurina, Marc Saez, Gemma Renart

**Affiliations:** 1 Research Group On Statistics, Econometrics and Health (GRECS), University of Girona, Girona, Spain; 2 CIBER of Epidemiology and Public Health (CIBERESP), Madrid, Spain; 3 Landscape Analysis and Management Laboratory, University of Girona, Girona, Spain

**Keywords:** Domestic violence, Police reports, Hurdle model, Spatial–temporal model

## Abstract

**Background:**

There are many studies that address the issue of gender-based violence, but few analyse the police-reported cases. It is as important to analyse the temporal and geographic distribution of these incidents as it is the sociodemographic profile of the accuser. To this effect, the present study aims to analyse the sociodemographic profile of the victims of the particular case of domestic violence that report the incident to the police and to evaluate the spatial–temporal distribution of these reports.

**Methods:**

Using the data from a database containing the police-reported incidents of domestic violence in Girona in the period 2012–2018, the risk of a police-reported incident was estimated by adjusting the two-part Hurdle model.

**Results:**

The risk of reporting incidents of domestic violence to the police is higher in the less deprived areas of the city, and the spatial distribution of these police reports corroborate this finding. Nevertheless, those areas with the greatest socio-economic deprivation were also the ones where there were less police reports filed. Also the prediction of less police reports in the census tract with the highest percentage of the population with an insufficient educational level coincides with the largest number of police reports made by women with a medium and university level education (56.1%) compared with 9.5% of police reports made by women with insufficient schooling.

**Conclusions:**

These results can be useful for social protection services to design policies specifically aimed at women residing in those areas with the highest risk. Moreover, the use of spatial statistical techniques together with geographic information systems tools is a good strategy to analyse domestic violence and other types of offences because they not only allow to graphically identify the spatial distribution, but it is also a good tool to identify problems related to this type of offence.

**Supplementary Information:**

The online version contains supplementary material available at 10.1186/s12889-022-12916-4.

## Introduction

Gender-based violence (GBV) was explicitly recognised in Spain following the official promulgation of a comprehensive law against gender violence in 2004 [1]. GBV is a global concept defined as violence directed towards women due to their being women and therefore considered by their aggressors as not having the minimum rights of freedom, respect, and decision-making capacity. However, it was not until the Council of Europe agreement on preventing and combatting violence against women, also known as the Istanbul Convention [2], presented in 2011 and in force in Europe since 1 August 2014 that the concept of the offence was broadened to include all forms of violence against women. That is including physical, psychological, and sexual violence, female genital mutilation, forced marriages, stalking, and forced abortion and sterilisation.

The offence of GBV is considered as a public offence, which can be prosecuted at the request of the victims themselves or a third party, or by private or public prosecution, given that according to article 106 of the Code of Criminal Procedures (LECRim) [3] “The act of committing a crime or an offence that gives rise to an ex-officio procedure does not expire at the withdrawal of the victim” in public offences. However, with some exceptions, any public offence must be reported to the police (Article 259) to be granted integral protection (Article 261). These offences are being introduced into the different European states that have signed and ratified it. In fact, it has currently been signed by 46 countries and ratified by 34 others, including Spain in 2014 [4].

There are many studies that have addressed the subject of GBV from different perspectives, mainly from the areas of health and education and aimed at determining the knowledge and attitudes of health professionals to be able to better detect and prevent cases of GBV [5–10], or at knowing the relationships between students or adolescents to facilitate and promote positive dynamics in their intimate, personal relations [11–13]. Most of these papers refer to violence against women, analysing its social and psychological impact on health from different perspectives. Regarding to health status point of view, women exposed to GBV have a greater likelihood of reporting poor self-perceived and mental health and more activity limitations [14]. From the origin perspective, inequalities were observed in the prevalence of GBV according to country of birth, with high prevalence in immigrant women [15]. From socioeconomical view, GBV shows variability among Autonomous Communities (CCAA) in Spain [16] and higher rates of GBV have been reported in CCAA with greater gender inequality [17]. Also, the lack of social support increases women’s vulnerability to being abused [18]. Some other works explore different general aspects of GBV, in a conceptual way. Thus, from a methodological perspective, GBV studies should always take into account the hierarchical and patriarchal social structure. Because this structure generates an unequal society that assigns roles to women that involve fewer opportunities for access to use of power [19]. However, few of them focus on the location and spatial pattern of reported crimes.

These latest ones, called “crime at place” studies analyze street corner, address, building or street segment crimes [20] and had contributed to the widespread adoption of place-based crime prevention measures such as environmental design, hot-spots policing, closed circuits television or surveillance intervention, among others. Although consistent findings that place-based prevention measures effectively reduce reported crime, little attention is given to the dark figures of crime, that is, that true crime is neither reported nor recorded by law enforcement agencies [21].

The official GBV statistics for Spain [22] show that there were 125,936 police reports made in 2019, a 24.5% decrease from 2018. Most of these were police statements with the formal status of a complaint made by the victim and represent the 69.8% of the total number of reported incidents during 2019. This reduction is surprising if we consider that figures for all police reported incidents have been rising since 2013.

In Catalonia in 2019, there were 16,946 police-reported cases of GBV, a 26.6% decrease from 2018. The overall trend follows that Spain as a whole, with a rising trend of police-reported cases of GBV since 2013. Focusing on the province of Girona, a similar pattern emerges. The number of reported cases in Girona in 2019 was 1,968, an 8.93% drop with respect to 2018.

Furthermore, the total number of deaths in Spain in 2019 due to GBV was 55 women, of which only 11 had previously reported the violence to the police, representing 20% of the total. These percentages, with a variation of between 19% and 31.8%, have been consistent for Spain since 2006, the first year the number of police reports were counted. In Catalonia, the number of deaths due to GBV in 2019 was 9 women, only one of which had previously made a police statement [22].

Regarding police-reported domestic violence, a particular case of GBV and on which this study is focused on, we found that the official data does not appear to accurately reflect the real number of cases of women that experience this situation. As Gracia E. [23] point out, financial dependency, shame, cultural beliefs, and fear [15, 19] are just some of the reasons why we are faced with an “iceberg” of domestic violence, with official data being just its tip and the submerged portion the large number of women in this situation who are invisible to society.

This means that using official crime statistics has two important implications. First, that there is a potential discrepancy between what victims believe to be a crime and what the police/office agencies actually record as a crime [24]. Second, little is known about dark figures in prevalent areas, which have several implications. First, they prevent understanding the factors that could inhibit this type of violence and, consequently, improve the authorities’ interventions. Second, an unreported gender-based violence reduces the likelihood the criminal will ultimately be convicted [25].

It appears that until now, the official police figures [22], the research that analyses the temporal and/or geographical distribution of police reports of gender-based violence, the studies that analyse the particular case of the relationship between domestic violence and the frequency of reported incidents and deaths, and other studies that analyse the association between police-reported intimate partner violence and femicide [18, 26, 27] have failed to explain the underestimation of the offence represented by the police reports files, as does the research into the sociodemographic profile of the people that suffer from this type of violence [28].

To this effect, Torrado [29] points out that general protection for women who suffer domestic violence would be more viable if the recommendation of the Committee of Ministers to Member States on the protection of women against violence were made effective, determining that protection will be given “whether or not the victim formally reports the offence” [30].

In this study, a database of municipal offences committed in the city of Girona in the period 2012–2018 was studied with the aim of knowing the sociodemographic profile of the victims of domestic violence that report the offence to the police, and to analyse the geographic patterns of these offences in order to contribute to revealing the dark figures of domestic violence.

## Methods

### Design

We used a longitudinal, ecological design where the dependent variable was repeatedly observed at different moments in time. The study population consisted of the population that resided in the different census tracts of Girona, Spain, between January 1, 2012 and November 6, 2018. The design was unbalanced, since the census tracts were not observed the same number of times.

The sample used in this study is completely anonymous, there is no information about sex, age, or any other socioeconomic characteristic that can help identify an individual. In fact, all the variables were contextual (none individual) observed at the census section level. The dependent variable, also contextual, was observed at the police section level. Using QGIS (version 2.18) [31], we join the attributes by location and assign the observations of the dependent variable to a census section.

### Variables

A description of the variables is given in Table 1. The dependent variable is the number of filed police reports of domestic violence that occurred during the period of study in the census tracts of Girona.

**Table 1:** Description of the variables

**Response variable**	
Cases census tract	Number of filed police reports of domestic violence that occurred in the census tracts of Girona in the period of study
**Explanatory variables**	
*Deprivation*	The Spanish Epidemiology Association’s deprivation index 2011 corresponding to each of the census tracts of Girona [32]. This index combines the information on six socioeconomic indicators (unemployment, manual workers, temporary workers, without compulsory schooling, without compulsory schooling young, without internet), calculated for each census tract and based on the data collected in the Spanish Population and Housing Census 2011 [33, 34]
*Unemployment*^a^	Percentage of unemployed population among active workers (above the age of 16) for each census tract of Girona
*Manual workers*^a^	Percentage of manual workers, i.e. employed workers in the building sector, in farming, among the employed or unemployed population that has worked previously, aged 16 or over for each census tract of Girona
*Temporary workers*^a^	Percentage of employed workers aged 16 and over in temporary employment among the employed or unemployed population that has worked previously, for each census tract
*Without compulsory schooling*^a^	Percentage of the population that did not complete compulsory schooling, i.e. people without studies and with an incomplete primary education
*Without compulsory schooling young*^b^	Percentage of the young population (16–29 years) that did not complete compulsory schooling, i.e. people with no qualifications and who had not completed primary education
Without internet^a^	Percentage of primary residences without access to the Internet
*Housing45*^a^	Percentage of homes in the municipal census tract of under 45 square meters
*Housing4560*^a^	Percentage of homes in the municipal census tract of between 45 and 60 square meters
*Foreign*^b^	Percentage of foreign people in the municipal census tract
*Foreign*_*Africa*_^b^	Percentage of Africans in the municipal census tract
*Foreign*_*SouthAmerican*_^b^	Percentage of South Americans in the municipal census tract
*Foreign*_*Asian*_^b^	Percentage of Asians in the municipal census tract

^a^data collected from the Spanish Population and Housing Census 2011[32]

^b^data collected from the INE[32]

Sociodemographic variables at the census tract level were considered as explanatory variables. The information was obtained from the 2011 census and from the National Statistics Institute (INE) [32].

The unemployment was categorized into quintiles, taking the first quintile (the one corresponding to census tract with the least unemployment rate) as the reference category. The deprivation index, the percentage of the population that did not complete compulsory schooling, and the percentage of the young population that did not complete compulsory schooling were also categorized into quintiles being the first three quintiles grouped in one category (which was the reference category for the deprivation index and the percentage of the population) and the two last quintiles in another (which was the reference category for the percentage of the young population).

### Statistical analysis

There is highly likely to be a selection bias in observational designs like the one we use in this study. Victims of domestic violence who report the offence are more likely to be observed and, therefore, to be present in the sample than those who are likewise victims but do not report the incident. In fact, there could be non-observed factors that intervene in the fact of making a police statement which would be correlated with the non-observable factors that affect the response variable [32]. In this case (known as endogenous selection), the standard weighting method (standardising) by age and sex would not correct the selection bias [32] and so a two-part model had to be used [33]. In the first part, the probability of a person being observed was estimated (in other words, having made a police report). These probabilities were then used as estimations in the second part of the model, with the aim of correcting the non-randomness, or in other words the selection bias.

These parts can be estimated separately. The problem with this approach, and in fact common to all multi-step approaches, is that the error inherent in the estimation committed in the first part is carried over into the second part. If this error is random the estimators are not biased, but they are inefficient. In other words, the confidence intervals will be very wide and it will be difficult to find statistically significant associations. However, if the error is not random, for example when the first part of the model is not well-specified, the estimators will be biased and their variances wrongly calculated. Therefore, following the approach proposed by Saez et al*.* [34], we decided to estimate the two parts together, specifying a two-part model called the Hurdle model. In the first part, we estimated the probability of a person being observed (i.e., of filing a police report), using a generalised mixed model (GLMM) with a binomial link. We included the variables that could explain that a person was observed as the variables associated with this probability.

The model for the first part was specified as follows:

Subject to the true risk in location ${x}_{i}$, the probability of a police report being filed in this location at moment t, $P\left({x}_{it}\right)$, $i=1,\dots , n;t=1,\dots ,T$, was distributed as a binomial.

$${Y}_{it}\left|P\left({x}_{it}\right)\right.\sim Binomial\left({n.trials}_{i},P\left({x}_{it}\right)\right)$$

${n.trials}_{i}$ is the population at risk of filing a police report in location ${x}_{i}$ at moment t. We used the population of the census tract [35].

The link function is as follows,

$$\mathrm{log}\left(\frac{P\left({x}_{it}\right)}{1-P\left({x}_{it}\right)}\right)={\beta }_{0}+{\eta }_{1i}+S\left({x}_{i}\right)+{{\tau }_{t}+\beta }_{1k} {Deprivation}_{ik}+\sum_{k=2}^{5}{\beta }_{2k} {Unemployment}_{ik}+{\beta }_{3} {Housing45}_{i}+{\beta }_{4} {Housing4560}_{i}+{\beta }_{5} {Foreign}_{i}+{\beta }_{6} {Foreig{n}_{Africa}}_{i}+{\beta }_{7} {Foreig{n}_{SouthAmerica}}_{i}+ {\beta }_{8} {Foreig{n}_{Asia}}_{i}+{\beta }_{9} {Manual workers}_{i}+{\beta }_{10} {Temporary workers}_{i}+ {\beta }_{11} {Without compulsory schooling}_{i}+{\beta }_{12} {Without compulsory schooling young}_{i}+ {\beta }_{13} {Without internet}_{i}$$

where the sub-index $i$ indicates the neighborhood in the city of Girona where the offence was committed; $t$ is the moment in time when the offence happened; and $\beta$ are the coefficients of the explicative variables (${e}^{\beta }$ is the relative risk associated with each of them). $\eta$, $\tau$ and $S$ are random effects. $\eta$ contains the non-spatially structured individual heterogeneity. In other words, it contains the non-observable confounders associated with each filed police report that do not vary over time. $\tau$ is a structured random effect over time containing the temporal dependency, and $S$ is a spatially structured random effect that is distributed normally with a zero mean and a Mátern covariance function:

$$Cov\left(S\left({x}_{i}\right),S\left({x}_{{i}^{\mathrm{^{\prime}}}}\right)\right)=\frac{{\sigma }^{2}}{{2}^{\nu -1}\Gamma \left(\nu \right)} {\left(\upkappa \Vert {x}_{i}-{x}_{{i}^{\mathrm{^{\prime}}}}\Vert \right)}^{\nu } {\mathrm{\rm K}}_{\nu } \left(\upkappa \Vert {x}_{i}-{x}_{{i}^{\mathrm{^{\prime}}}}\Vert \right)$$

where ${\mathrm{\rm K}}_{\nu }$ is the modified Bessel function of the second type and order $\nu >0$. $\nu$ is a smoothing parameter, ${\sigma }^{2}$ is the variance, and $\kappa >0$ is related to the range ($\rho =\sqrt{8 \nu }/\kappa$), the distance to which the spatial correlation is close to 0.1.

In the second part, we use a GLMM with a Poisson link to model the number of police reports filed in a census tract. Notably, we use this link and not a truncated Poisson like in Saez et al*.* [34] because the number of census tract where no police reports were filed is very small. We included the same explanatory variables as in the first part of the model.

Regarding the second part, its specification was as follows:

Subject to the true risk in location ${x}_{i}$, the mathematical expectation of police reports being filed in the census tract, $\theta \left({x}_{it}\right)$, $i=1,\dots , n$; t = 1,…,T, was distributed as a Poisson.

$${Y}_{it}\left|{\theta }_{it}\right.\sim Poisson\left({\theta }_{it}\right)$$

In this case, the link function was as follows,

$$\mathrm{log}\left({\theta }_{it}\right)={\beta }_{0}+{\eta }_{1i}+S\left({x}_{i}\right)+{{\tau }_{t}+\beta }_{1k} {Deprivation}_{ik}+\sum_{k=2}^{5}{\beta }_{2k} {Unemployment}_{ik}+{\beta }_{3} {Housing45}_{i}+{\beta }_{4} {Housing4560}_{i}+{\beta }_{5} {Foreign}_{i}+{\beta }_{6} {Foreig{n}_{Africa}}_{i}+{\beta }_{7} {Foreig{n}_{SouthAmerica}}_{i}+ {\beta }_{8} {Foreig{n}_{Asia}}_{i}+{\beta }_{9} {Manual workers}_{i}+{\beta }_{10} {Temporary workers}_{i}+ {\beta }_{11} {Without compulsory schooling}_{i}+{\beta }_{12} {Without compulsory schooling young}_{i}+ {\beta }_{13} {Without internet}_{i}$$

With the definition of the parameters, the variables, and the random effect the same as previously.

### Representing relative risks on a map

Geographical Information Systems (GIS) [31] were used to map the census tract were each victim lived to identify hotspots in the study area. Hotspots are areas with the highest concentration of domestic violence victims, appearing in a dark color on the maps. We represented the relative risks (RRs) estimated in the models on a map of the region under study (i.e. Catalonia) to evaluate the possible existence of a geographical pattern in the distribution of the offences. The maps at the census tract levels were obtained from the INE [32]. We also computed exceedance probabilities, which are the probabilities that the smoothed relative risks were above 1 [36]. Richardson et al*.* recommend using the cut-off of 80% (and 20%) as a specific interpretation rule. To this effect, when the exceedance probability is greater than 80% (less than 20%), a reasonable sensitivity will be achieved. This cut-off can be used as a measure of statistical significance of the smoothed risk and as a way of helping to assess the existence of agglomerations of excess cases (i.e. clusters). The exceedance probabilities were also represented on a map of the study area. Given the complexity of our model, we decided to perform inferences using a Bayesian framework. More specifically, we followed the Integrated Nested Laplace Approximation (INLA) approach [37], within a (pure) Bayesian framework. All the analyses were made using the free software R (version 3.6.2) [38], through the INLA package [37, 39]. The maps were represented in QGIS (version 2.18) [31].

## Results

**Fig. 1:**
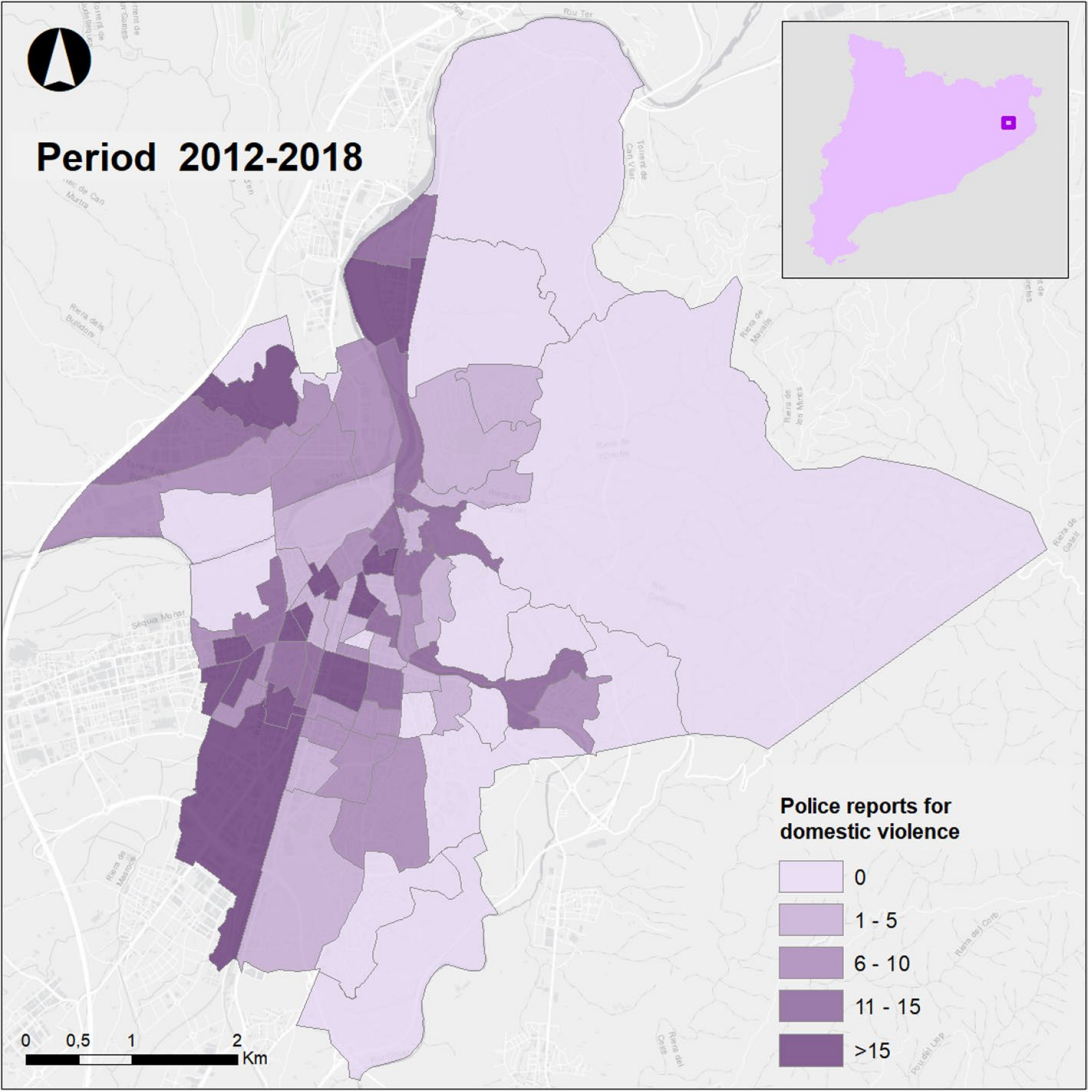
Police reports of domestic violence in the census tracts of Girona during 2012–2018

The number of filed police reports of domestic violence that occurred in the census tracts of Girona in the period of study are showed in Fig. 1. It is shown that the area with ore complaints is the central and western part of the municipality of Girona.^1^

The descriptives of the variables used are given in Table 2. All the variables are expressed in percentages except for the deprivation index. First, we can observe the mean percentages for the homes with the least number of square metres in the set of census tract. The maximum percentage of homes of 60 square metres or less is 27.74%. Regarding the percentages of foreigners, some figures that stand out are the small mean percentage of 1.54% of Asian people and the large variability in the overall percentage of foreign people, oscillating between 0 and 53.50% in the various census tracts. Regarding the level of schooling of the general population, the mean was 30.3%, corresponding to the 40% of the census tract with the lowest educational level (Q4 + Q5). When we calculated the level of schooling of the young people, we saw that in 60% of the census tract with the most schooling (Q1 + Q2 + Q3) there was an average value of 13.81% of insufficient schooling.

**Table 2:** Descriptives

	**Mean (standard deviation)**	**Min/Max**
Deprivation	-0.62 (0.73)	-2.26 / 1.67
Deprivation Q4-Q5	0.77 (0.014)	-0.53 /1.67
Unemployment	24.46 (9.47)	2.38 / 44.76
Q1	12.12 (1.91)	2.38 / 14.46
Q2	17.07 (1.80)	14.73 /20.10
Q3	23.36(1.27)	21.65 /25.89
Q4	28.10 (2.43)	26.24 / 33.66
Q5	38.89 (3.75)	34.46 /44.76
Housing45	4.25 (5.82)	0.00 / 16.48
Housing4560	10.68 (5.12)	0.95 / 27.74
Foreign	21.51 (15.46)	0.00 / 53.50
Foreign_Africans_	6.73 (7.28)	0.00 / 24.27
Foreign_SouthAmerican_	12.98 (10.38)	0.00 / 44.63
Foreign_Asian_	1.54 (3.56)	0.00 / 12.71
Manual workers	52.24 (17.42)	7.32 / 90.51
Temporary workers	24.84 (8.27)	5.26/ 47.14
Without compulsory schooling Q4-Q5	30.13 (0.35)	20.59 / 63.64
Without compulsory schooling Young Q3 or less	13.81 (0.20)	7.89 / 20.58
Without Internet	33.38 (9.73)	12.90/ 65.96

There was also a large variability in terms of the manual workers, with a mean percentage of 52.24% up to maximum percentages of up to 90.51%. Regarding casual employees, the average percentage is much lower, with a reported average value of 24.84%. The deprivation index values were not expressed in percentages and we showed the values for both the global index and the most deprived census tract corresponding to the higher quartiles.

Last, there is the average percentage of unemployment of 24.46%, oscillating between 2.38 and 44.76%, and the values corresponding to the quintiles into which we have broken down the index.

The results for the fit of the models are shown in Table 3. In the first model, we only show the significance of the index, which indicates that the more deprivation the fewer the police reports filed for domestic violence. In the second model, where we adjust for the index variables and also the rest of the variables individually, as specified in the model, we obtained estimates which their 95% credibility intervals did not contain the unity for the homes with the least square metres and for the foreigners and their different geographical origins. The direction of significance is that in the census tract where the percentage of small homes is greater, there are also less reports filed. The interpretation in the case of the percentage of foreigners is variable. For the census tract with a greatest percentage of people from Africa and South America there are less reports filed, but in the census tract with the largest global percentage of foreigners and where there are more people from Asia, there are more police reports filed.

**Table 3:** Fit of the models

	**OR**	**95% credibility interval**
Deprivation^a^		
Q_4_ and Q_5_	0.573	(0.411, 0.798)^b^
**Unemployment**		
Unemployment_Q2_	1.009	(0.688, 1.484)
Unemployment_Q3_	1.196	(0.675, 2.117)
Unemployment_Q4_	0.846	(0.487, 1.459)
Unemployment_Q5_	0.994	(0.515, 1.884)
**Housing45 (%)**	0.894	(0.836, 0.953)^b^
**Housing4560 (%)**	0.995	(0.976, 1.014)
**Foreign (%)**	1.028	(1.007,1.050)^b^
**Foreign**_**Africans**_** (%)**	0.976	(0.938, 0.998)^b^
**Foreign**_**South american**_** (%)**	0.978	(0.955, 1.002)^b^
**Foreign**_**Asian**_** (%)**	1.146	(1.060, 1.241)^b^
**Manual workers**	1.007	(0.995, 1.020)^a^
**Temporary workers**	1.000	(0.983, 1.018)
**Not completed compulsory schooling**		
Q4 and Q5	0.787	(0.528, 1.165)^a^
**Not completed compulsory schooling young**		
Q3 or less	0.887	(0.610, 1.273)
**Without Internet (%)**	0.991	(0.975, 1.008)^a^

^a^The 90% credibility interval did not contain the unity

^b^The 95% credibility interval did not contain the unity

We also saw estimates which their 90% credibility intervals did not contain the unity for the variable manual workers, representing the census tract with the highest insufficient level of education, and indicating the percentage of people without Internet. The interpretation of the results obtained is that the greater the percentage of manual workers, the larger the number of police reports filed, and that in census tracts with the highest percentage of people with an insufficient level of education and with more people without Internet, there are less police reports of domestic abuse filed.

**Fig. 2:**
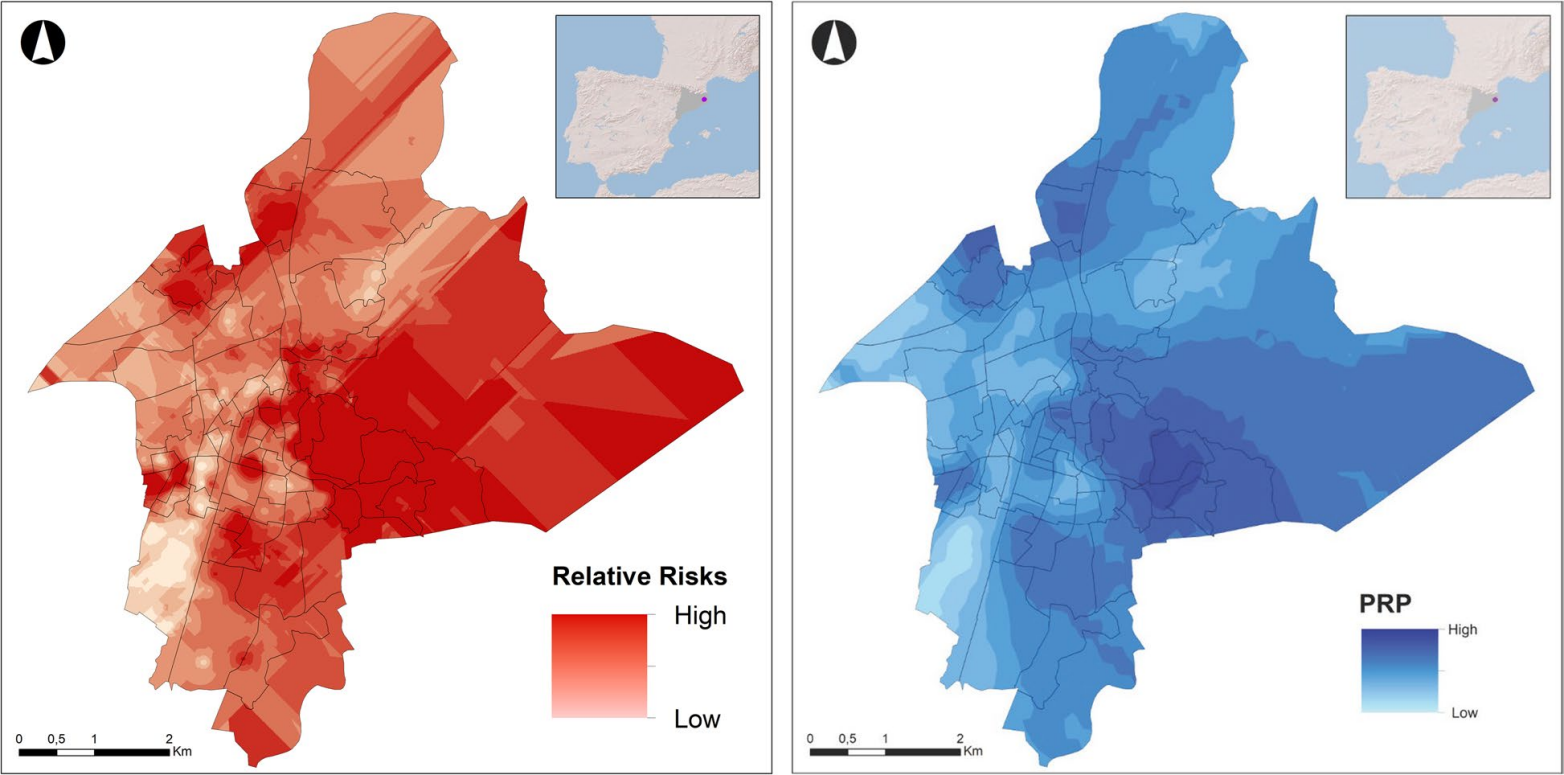
Maps Left: relative risk (RR) of a police report of domestic violence being filed. Right: probability (PRP) of the relative risk being more than the unit

Last, Fig. 2 shows the maps. The relative risk (RR), or the probability of a police report of domestic violence being filed, not explained by the model is shown in the left-hand part of the model, while on the right is the assigned conditional probability after observing an incident, showing the probability of the relative risk being more than the unit and, therefore, more than its significance.

More specifically, the RR map shows the areas with the highest risk of a police report being filed, or in other words the areas where there are likely to be problems of domestic violence but these incidents have not been reported to the police as we can see in the Fig. 1. The areas that stand out in the first map are first the area in the east where the deprived census tract with a large number of immigrants are located; an area in the west with two smaller nucleuses; the nucleus in the north-east and another small nucleus a little further to the south; a nucleus in the southern part; and last, a small area in the central part with a more heterogeneous population and therefore difficult to classify.

The map that shows the PRP helps to interpret the results provided by the map of relative risks, in the sense that it gives us geographical patterns that show us the areas or groups of areas with the highest risk of there being police reports of domestic violence, i.e. hotspots. This second map clearly shows the four high-risk areas: the area in the north-west with 34.20% of the population with an insufficient level of education compared with the average of 18.98% for the set of neighbourhoods, a percentage of 44% of houses without Internet compared with the global average of 33.38%, a percentage of smaller houses of 5.59% compared with the 4.25% global average, and a foreign population percentage of 26.15% compared with the global 21.51%.

The small western area located more towards the south also appears in this second map and is characterised by 35.80% of the population being made up of foreigners versus a global average of 21.51. The percentage of Africans stands out at 7.20% versus a global average of 6.73%, in addition to 27.73% with an insufficient level of education compared with the global 18.98%, and a high percentage of small houses of 16.48% versus the global 4.25%. This area is also characterised by a high percentage of immigrants from Asia, representing 12.71% of the population compared with a global average of 1.54%.

The eastern zone is much more clearly depicted in this second map, with an obvious south-eastern area characterised by 35.80% of immigrants compared with the global average of 6.73%. A total of 61.49% of homes in this area are also without Internet compared with the 33.38% global average, and 42.81% of the population have an insufficient level of education, which is way above the global average of 18.98%.

The darker area in the south stands out for its high percentage of foreigners, at 53.50% compared with the global 21.51%. There is also a high percentage of South American immigrants, at 40.64% compared with the global 12.98%, and a percentage of the population with an insufficient level of education of 21.49% compared with a global 18.8%.

Last, the small central zone shown on the map also has higher than average percentages of foreign immigrants (25.54%, compared with a global 21.51%), African immigrants (9.64% compared with a global 6.73%), and homes without Internet (35.79%, compared with a global 33.38%).

## Conclusions and discussion

Using a database of police reports of domestic violence in the city of Girona, a sociodemographic profile analysis was made of the victims, enabling us to know the main factors that impact on the fact of reporting this type of criminal offence. The modelling of the relative risk of making a police report based on a two-part model (which resolved the possible risk of selection and the problem of accumulation of errors) together with the geographic information systems (GIS) enabled us to affirm that there was a spatial pattern to the police reports of domestic violence made in the different census tracts in Girona, in the same way as geographic variability was found in other previous studies [15, 40] depending on the predominant social and demographic characteristics of each area.

Generally, and as a main conclusion of this study, we observed that the areas with the greatest socio-economic deprivation were also the ones where there were less police reports filed. This conclusion was supported by the fact that in the census tracts with higher percentages of small houses, a population with an insufficient level of education, homes without Internet, and a population of African and South American origin, there were less police reports of domestic violence filed. It is known that the reluctance of victims of domestic violence to report incidents has a strong socioeconomic and cultural component that makes the victim unable to make a new life away from their aggressor [41].

Many victims believe that when they report the domestic violence and remove themselves from their aggressor, they will not be able to get by on their income only. This situation is further aggravated when the victim has no family support or they are women that have never worked outside the home, either because they have been keeping the house and looking after the children, or because their partner did not let them work [42]. Other times it is the aggressor that is financially dependent on the victim, who often justifies the aggressor’s behaviour or feels guilty about the situation, leading them to feel unable to abandon their aggressor [43].

In the case of women of immigrant origin, other beliefs linked to the legal and political system in this country must be added to the mix. While it is true that our model shows that the greatest number of police reports for domestic violence are made in the census tract with the highest percentage of foreign population, we also observe that this immigrant population does not behave homogenously. Whether it is because they believe they will be deported from the country or because they perceive more obstacles to accessing and making use of legal resources [28, 44], women of African and South American origin are less inclined to report domestic violence to the police [45–47].

Last, there are family and social pressures that influence reluctance to file a police report for domestic violence, and which are linked to the self-esteem of women victims [27, 48, 49]. Sometimes it is the family themselves, or the aggressor’s family, who justifies the behaviour; other times it is the social environment wherein the aggressor is seen as a “good person”, a “good father” or a “hard working man”; or it could simply be that the victim perceives the situation as a personal failure for not having chosen a better partner. As pointed out [50, 51], a persons’ self-esteem is tightly related to their educational level and so it comes as no surprise that the prediction of less police reports in the census tract with the highest percentage of the population with an insufficient educational level coincides with the largest number of police reports made by women with a medium and university level education (56.1%) compared with 9.5% of police reports made by women with insufficient schooling [52].

Despite one of the limitations of this study being the use of data provided by the municipal police in a specific section, the methodology employed enabled us to identify the factors that impact on there being more police reports for domestic violence in the hotspots where there had been hardly any reported domestic violence. To this effect, these results could be useful for social protection services to design policies specifically aimed at women residing in ‘hotspot’ neighbourhoods. This same methodology could also be used to detect hotspots of other types of risks. By way of example, it could be used as a support for a warning system for epidemics to help prevent outbreaks, or even for pandemics such as COVID-19. The use of spatial statistical techniques together with GIS tools could help to combat domestic violence and other types of offences because they not only help us to create colour maps to establish spatial patterns, but they also enable us to identify problems related to this type of offence. This type of work should help the municipalities to visualize the problem and take actions at the city and neighbourhood level.

## Supplementary Information

**Additional file 1:** **Supplementary Figure 1.** Police reports of domestic violence in the census tracts of Girona per year (2012-2018).

## Data Availability

The datasets generated and or analysed during the current study are available upon request to the corresponding author.

## Footnotes

1 Annual case maps are available as a supplementary material.
