# Supplementary material for: Analysis of the geographic pattern of the police reports for domestic violence in Girona (Spain)
Source: BMC Public Health. 2022 Mar 21;22:552. doi: 10.1186/s12889-022-12916-4 (PMC8935101; doi:10.1186/s12889-022-12916-4)
Supplement: Supplementary file 1 — Additional file 1: Supplementary Figure 1. Police reports of domestic violence in the census tracts of Girona per year (2012-2018). [file 12889_2022_12916_MOESM1_ESM.pdf]

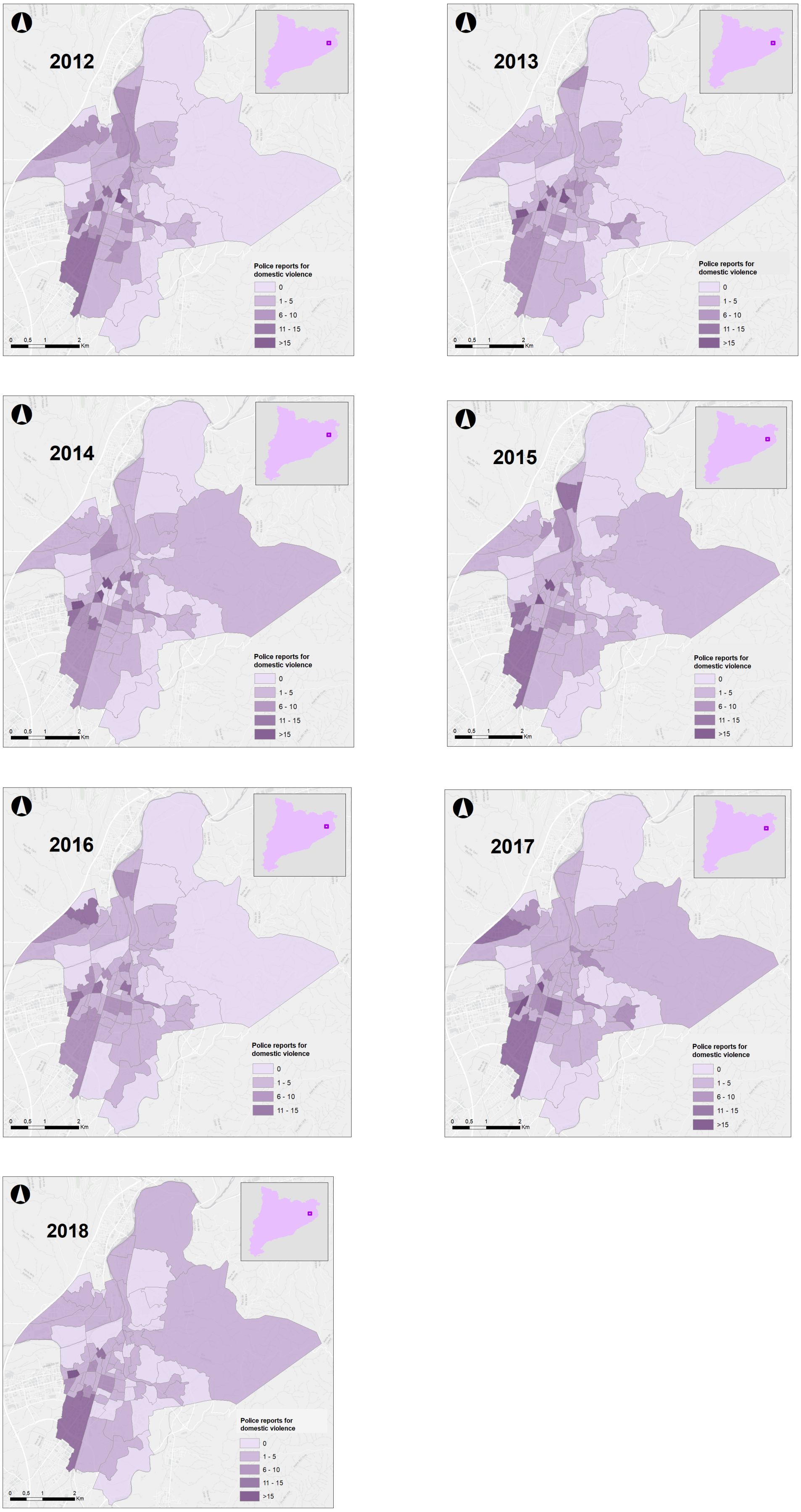

Supplementary Figure 1. Police reports of domestic violence in the census tracts of Girona per year (2012-2018)
